# More robust detection of motifs in coexpressed genes by using phylogenetic information

**DOI:** 10.1186/1471-2105-7-160

**Published:** 2006-03-20

**Authors:** Pieter Monsieurs, Gert Thijs, Abeer A Fadda, Sigrid CJ De Keersmaecker, Jozef Vanderleyden, Bart De Moor, Kathleen Marchal

**Affiliations:** 1 ESAT-SCD/SISTA, K.U. Leuven, Kasteelpark Arenberg 10, 3001 Leuven-Heverlee, Belgium; 2 Centre of Microbial and Plant Genetics, K.U. Leuven, Kasteelpark Arenberg 20, 3001 Leuven-Heverlee, Belgium

## Abstract

**Background:**

Several motif detection algorithms have been developed to discover overrepresented motifs in sets of coexpressed genes. However, in a noisy gene list, the number of genes containing the motif versus the number lacking the motif might not be sufficiently high to allow detection by classical motif detection tools. To still recover motifs which are not significantly enriched but still present, we developed a procedure in which we use phylogenetic footprinting to first delineate all potential motifs in each gene. Then we mutually compare all detected motifs and identify the ones that are shared by at least a few genes in the data set as potential candidates.

**Results:**

We applied our methodology to a compiled test data set containing known regulatory motifs and to two biological data sets derived from genome wide expression studies. By executing four consecutive steps of 1) identifying conserved regions in orthologous intergenic regions, 2) aligning these conserved regions, 3) clustering the conserved regions containing similar regulatory regions followed by extraction of the regulatory motifs and 4) screening the input intergenic sequences with detected regulatory motif models, our methodology proves to be a powerful tool for detecting regulatory motifs when a low signal to noise ratio is present in the input data set. Comparing our results with two other motif detection algorithms points out the robustness of our algorithm.

**Conclusion:**

We developed an approach that can reliably identify multiple regulatory motifs lacking a high degree of overrepresentation in a set of coexpressed genes (motifs belonging to sparsely connected hubs in the regulatory network) by exploiting the advantages of using both coexpression and phylogenetic information.

## Background

Several motif detection algorithms have been developed to discover overrepresented motifs in sets of coexpressed genes (for instance [1-8]). The rationale behind these methodologies is that a set of genes regulated by the same transcription factor should contain in their intergenic regions motifs that are statistically overrepresented as compared to their occurrences in unrelated sequences. These methodologies have proven to be useful in many applications (for instance [9-12]). However, usually their performance rapidly drops as the signal to noise ratio (defined as the number of sequences that actually contain the motif versus the number of sequences lacking the motif) in the data set decreases [13,14]. This drop in performance is also evident in an assessment study of 13 different computational *de novo *motif detection tools, where the number of correct motifs retrieved was very low in sets of noisy data [15]. However, noisy data is mostly the case encountered in sequence sets derived from genome wide expression profiles, such as microarrays. The high noise level in such sequence sets comes as a result of the processing of the microarray data (for instance, the filtering and clustering procedures used), as well as the nature of the biological processes in the cell itself. For instance, when comparing mRNA expression between a wild type and a regulator knock out strain, besides the direct targets of the regulator, indirect targets are also affected in their mRNA expression in the knock out. In such mutants the complete regulatory network acting downstream of the mutated regulator is disturbed. Lists of genes derived from these experiments contain targets of more than one regulatory protein lowering the relative overrepresentation of a particular motif

In order to get over this problem, we have developed an approach that exploits orthology information besides coexpression, to discover *de novo *motifs. Orthology information is introduced by phylogenetic footprinting which is based on the assumption that among phylogenetically related species, the regulating sequences in the upstream regions of orthologous genes are selectively conserved by evolution. Phylogenetic footprinting has been used for the detection of motifs with relative success [16-23]. In these approaches, however, the orthology information is only used within a set of orthologous intergenic regions to create motif models. Recently, a number of algorithms were developed that permit for the mutual comparison of the motif models derived from different sets of orthologous intergenic regions, followed by the clustering of the conserved regions that share the same regulatory motif. In one approach, Jensen *et al*. [24] applied phylogenetic footprinting using Gibbs Sampling and then grouped the conserved regions using a Bayesian clustering algorithm. A similar way of clustering motif models derived from phylogenetic footprinting was developed by Qin *et al*. [25] and Van Nimwegen *et al*. [26]. Another approach is developed by Wang and Stormo [27], where conserved regulatory regions were detected using Wconsensus [5] and where the regions sharing the same regulatory motif were clustered by gradually merging motif models of different orthologous sets (PhyloCon). In their recent paper, Wang and Stormo [28] used a different clustering approach; they would first align the models and consequently cluster them according to their alignment scores (PhyloNet). Our methodology is a combination of Gibbs Sampling-based phylogenetic footprinting, with two-step clustering (first aligning motif models, then clustering based on the alignment score). For phylogenetic footprinting, we developed a new algorithm called BlockSampler, which is an extension of the Gibbs Sampling-based MotifSampler [2], optimized towards phylogenetic footprinting. For the alignment of the different motif models returned from BlockSampler, we developed a second algorithm called BlockAligner, which aligns matrices describing conserved regions using a Smith-Waterman approach [29].

The methodology based on these two new algorithms is capable of detecting motifs with weak overrepresentation in a set of coregulated genes. By applying our procedure on gene lists derived from real genome wide expression studies, we show its ability to function effectively in noisy data. In this context, the use of orthology information compensates for a lower degree of motif overrepresentation We also compared its performance on a test data set, with that of two other motif detection tools: one that is exclusively based on coexpression information, AlignACE [7], and another that is most similar to our method, PhyloCon [27]. With this comparison, we demonstrated the robustness of our method over the two mentioned. Using our method on two real biological data sets proved its biological applicability.

## Results

### General strategy

Several motif detection algorithms have been developed to discover overrepresented motifs in sets of coexpressed genes. However, in a noisy data set the motif might not be sufficiently overrepresented to allow detection by classical tools. To be able to recover motifs in such context, we developed a procedure in which we first use phylogenetic footprinting to delineate all potential motifs in each gene. Then, we mutually compare all detected motifs and identify the ones that are shared by at least a few genes in the data set as potential candidates.

**Figure 1:**
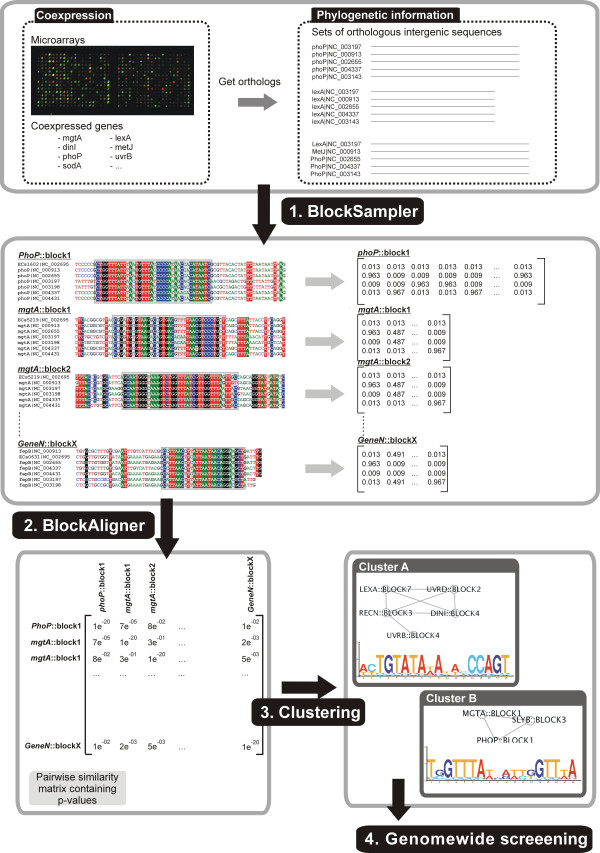
**Flowchart of our algorithm**. Input data: Based on microarray data, coexpressed genes are identified. For each of these coexpressed genes, the orthologous intergenic sequences are identified. Step 1: BlockSampler: within a set of orthologous intergenic sequences, conserved blocks are detected and stored as a Position Specific Scoring Matrix (PSSM). Step 2: BlockAligner: The resulting PSSM's corresponding to conserved blocks, are aligned to each other with BlockAligner, which is a local alignment tool that assigns a p-value to each alignment as a quality measure. Step 3: based on all these pairwise comparisons and their corresponding p-values, a clustering is performed to group intergenic regions with a similar regulatory motif. Based on the BlockAligner results, a regulatory motif model is constructed for each cluster. Step 4: the detected motif models are used to screen all intergenic regions that were the input for the methodology.

The complete analysis flow is represented in figure 1 and consists of the following steps: starting from a list of differentially expressed or coexpressed genes, we find the orthologs for each gene in a number of closely related species. The obtained data sets, consisting of the intergenic sequences of orthologous genes, are subjected to phylogenetic footprinting using a Gibbs Sampling tool called BlockSampler (Step 1). This yields a list of conserved regions (blocks) for each of the orthologous data sets, corresponding to all potential motifs. Searching for the motifs shared by at least some of the original list of coexpressed genes, we mutually align our blocks with BlockAligner (step 2), and then we construct a multiple alignment using the pairwise alignment scores to delineate further the potential regulatory motifs (step 3). As our methodology only detects motifs in genes for which orthologous information is available, some motif hits in the initial gene list might escape detection. To recover these motifs, we use the motif models we obtained from the previous step to screen the intergenic sequences of the remaining genes (step 4).

Here we give an overview of the main tools we used in our flow. Algorithmic details can be found in the methods section.

BlockSampler is used to detect conserved motifs in intergenic sequences of orthologous genes. The algorithm is an adapted version of MotifSampler, a motif detection algorithm based on Gibbs sampling [2]. Due to the specificities of the scoring scheme (see methods) the algorithm does not require the motif to be present in each single orthologous sequence. Although regions conserved in the entire set of sequences will receive a better score, the regions conserved in less than the entire set could still be retained. In contrast to the original implementation [30], BlockSampler allows for scoring each orthologous intergenic sequence in the input data set with its corresponding species specific background model. Since it is often the case with closely related species that big stretches around the motifs are conserved [23,31], BlockSampler was adapted to look for long conserved sequence blocks. BlockSampler also allows for the choice of a reference species, usually corresponding to the species of interest for which the experimental data are available. The consequence of using a reference species is that only the conserved regions that include the reference species will be retained.

BlockAligner performs a local ungapped pairwise alignment, based on dynamic programming for the mutual comparison of sequence blocks, represented as frequency matrices. The scoring scheme of BlockAligner is based on the Kullback-Leiber distance. We used a graph clustering algorithm to group the blocks shared by multiple genes [32]. This algorithm is optimized to cluster sequences based on pairwise alignment scores.

### Evaluation of the analysis flow on test data

**Figure 2:**
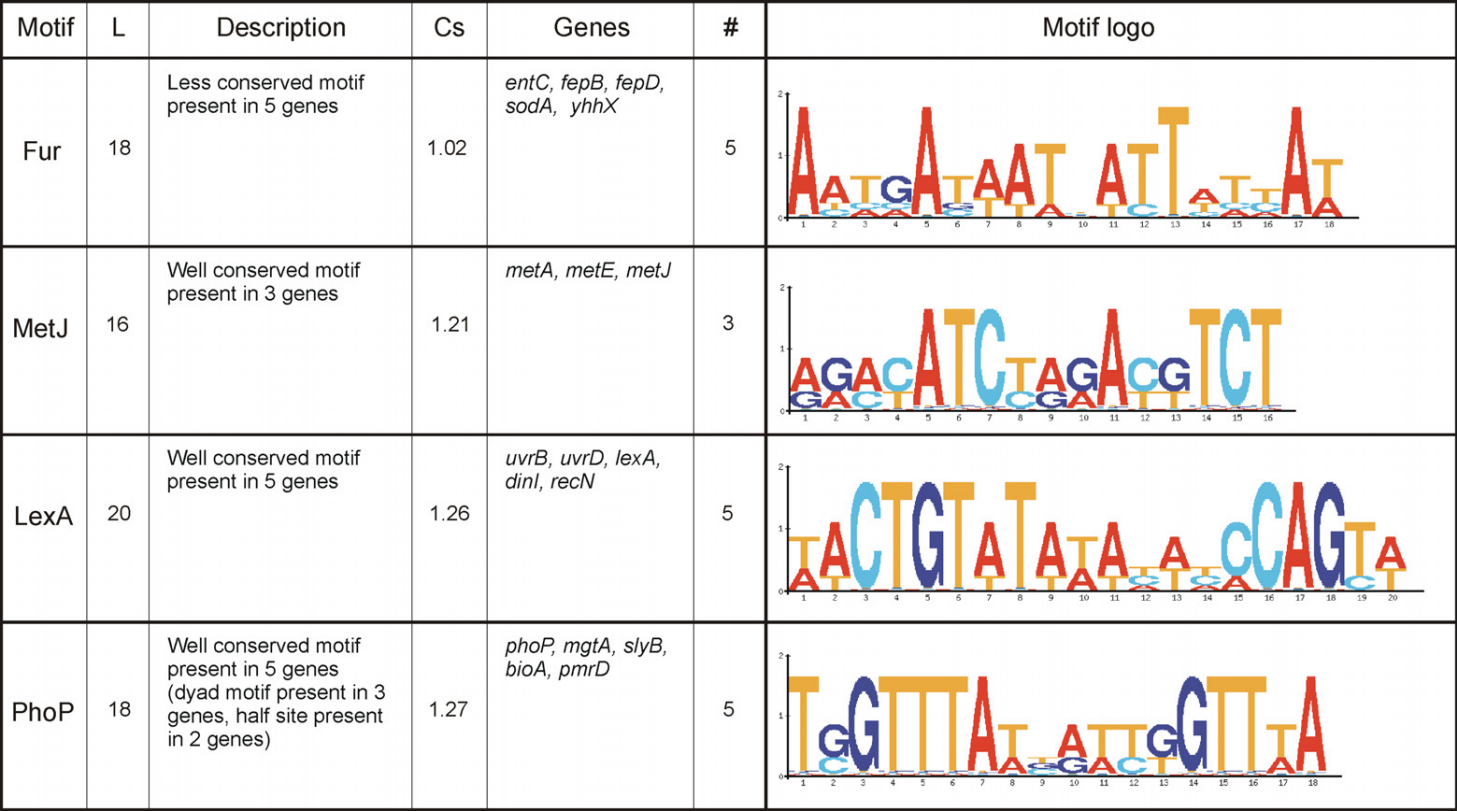
**Composition of test data sets**. Detailed information about the test data set. For each motif, the genes are shown of which the intergenic region is used in the test data set. L gives the length of the motif model. Cs indicates the consensus score of the motif model created based on the instances in these genes; # indicates the number of genes that contain the corresponding motif; the motif logo gives a visual representation of the information content of the motif model [48].

We applied our analysis flow on test data sets with known motif instances. As shown in figure 2, the data sets are made up of sequences with instances for the motifs PhoP, LexA, Fur and MetJ, imbedded in a varying number of random sequences. Five collections of data sets were created with an increasing number of random sequences, decreasing the signal to noise ratio from 18- 7% for Fur and LexA and 11- 4% for MetJ and PhoP (dyad motif). For the purpose of evaluating the performance of the methodology, we adopted four measurements: recovery rate, number of false positives, specificity and sensitivity. The former two are a reflection of how well a true motif model is extracted, while the latter two are measurements of the extracted motif's quality. Specifically, the recovery rate measures the number of times a motif model corresponding to a true motif is recovered by the algorithm; the number of false positives measures the average number of motif models not corresponding to the true motifs recovered by the algorithm; the sensitivity measures the ratio of true motif instances that contributed to the recovered motif model; the specificity measures the ratio of false motif instances that contributed to the recovered motif model (for details see methods). Note that these values were calculated before the last screening step of the algorithm, so that all instances recovered by screening are not included in the calculations.

**Figure 3:**
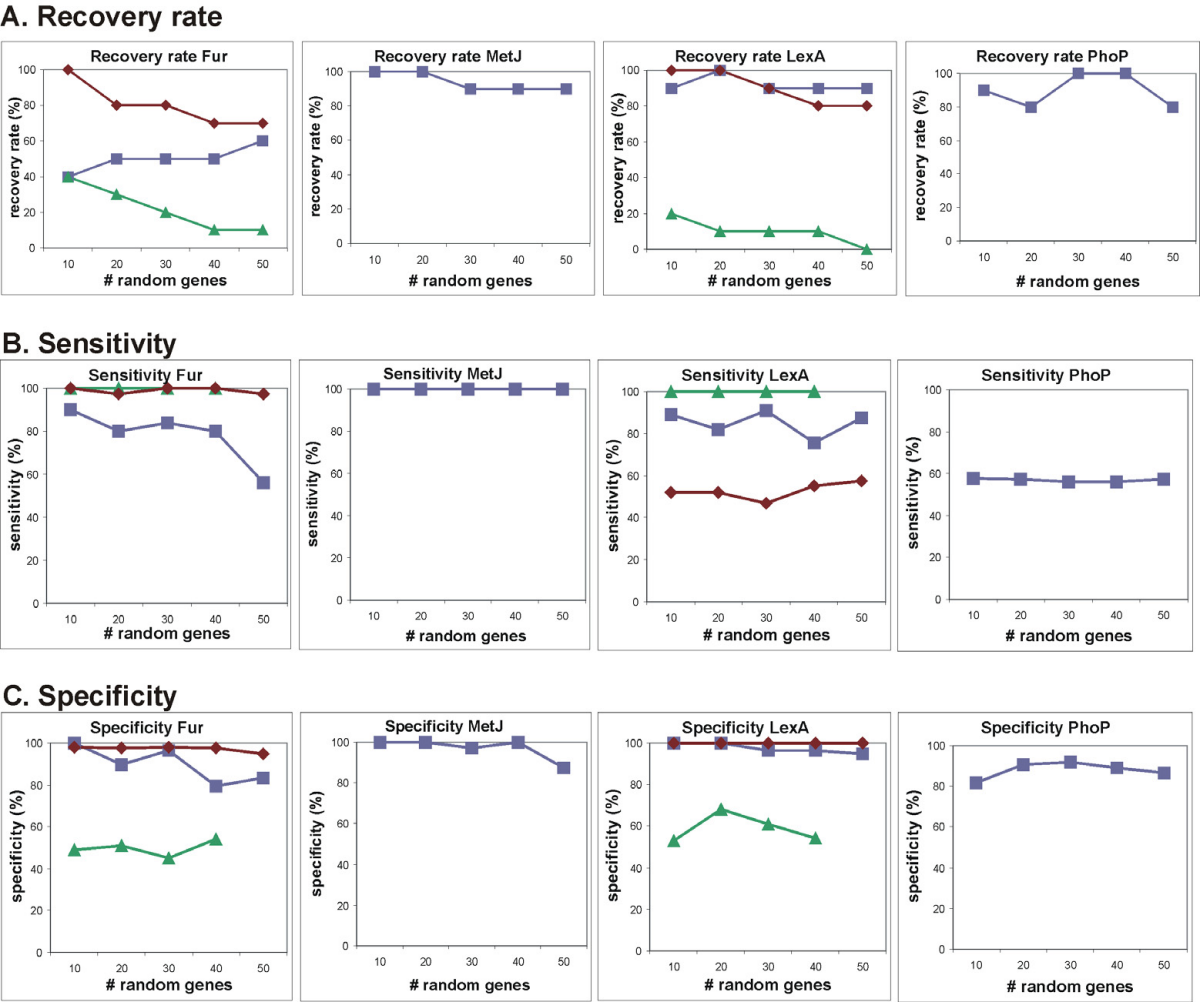
**Plot of the recovery rate, sensitivity and specificity**. For each of the four motifs (Fur, MetJ, LexA and PhoP), the recovery rate (A), the sensitivity (B) and specificity (C) is plotted for each of the three algorithms (BlockSampler: ■, AlignACE: ▲, PhyloCon: ◆). At the X-axis of each plot, the number of added random genes is indicated. No sensitivity or specificity data for a specific algorithm means that this characteristic could not be calculated due to a recovery rate of 0.

As shown in figure 3 and the supplementary table 1[33], our methodology performed well in retrieving the MetJ, LexA and PhoP motif models, with relatively low effect of the increasing noise in the data set. Only for the Fur motif the recovery rate was low, but with high resistance to noise. Analysis of the results showed that during the run of the methodology, the Fur motif instances were clustered in a single cluster (step 3), however, together with degenerated blocks lacking Fur instances. The presence of such blocks in the subsequent motif delineation step causes the consensus score of the Fur motif model to drop below the selected threshold, explaining why it was not frequently recovered using our stringent criteria.

The number of false positives picked up by the algorithm was low (table 1), ranging between an average of 0.5 in the low noise data sets to 0.8 in the high noise sets. This shows the robustness of the algorithm against noise in the data.

Regarding the quality of the motif models recovered, figure 3 shows that the algorithm achieved relatively high sensitivity and specificity for Fur, MetJ and LexA, indicating that most true instances contributed to the model, and that few false ones did. In the case of PhoP, the sensitivity was low due to the fact that the model recovered by our algorithm was the dyad variant, while the two instances of the half site variant (*bioA *and *pmrD*) did not contribute to the model.

### Importance of the selected species

To assess the importance of adding a distantly related species to the analysis (in our case *Y. pestis*), we tested to what extent motif instances in this species contributed to the motif models returned by our algorithm. Instances of *Y. pestis *seemed to have contributed to the resulting motif models in more than 90% of the cases for MetJ and LexA, more than 80% for PhoP, and more than 75% for the Fur motif. On the other hand, adding *Y. pestis *did not decrease the sensitivity of our motif models: motif instances that were only conserved in *S. typhimurium *and *E. coli*, but not in *Y. pestis *were also recovered. These results indicate that aligned blocks are not dominated by the sequences of the most related species and that adding a distantly related species helps delineating the true motifs without decreasing the sensitivity, even when motif instances are no longer conserved across all species.

### Comparison with other methodologies

We compared the performance of our methodology to that of two other motif detection algorithms: AlignACE, a Gibbs sampling algorithm designed to detect overrepresented motifs in a set of coregulated genes and PhyloCon, an algorithm that uses information on coexpression and orthology for motif detection.

AlignACE was applied to our test data sets without taking orthology information into account. Execution parameters are described in Methods. As displayed in Figure 3, only two out of the four motifs could be detected by AlignACE with very low recovery rates. In addition, the performance for those two motifs drops significantly when increasing the number of random genes (Figure 3 panel A). While the number of false positives seems to be independent of the noise, it is clearly high (varying between 16.9 and 18.3 compared to 0.5–0.8 for our algorithm (Table 1)). Regarding the quality of the motifs recovered, Fur and LexA, the sensitivity was always high, however, at the expense of a lower specificity. No values for sensitivity and specificity were calculated for PhoP and MetJ as these motifs were not detected. Our methodology clearly outperformed AlignACE in three aspects: the number of true motifs recovered, the quality of the motifs and the robustness against noise. This result is expected as AlignACE uses one source of information i.e. genes from one species, while our methodology depends on two sources i.e. single species genes and their orthologs.

In the case of PhyloCon, it also failed to recover MetJ and PhoP (figure 3). Although it was previously reported that it is possible to detect the MetJ motif in a dataset containing only MetJ intergenic regions [27] (confirmed in our lab), PhyloCon clearly failed to do so in the noisy context of our study. For LexA, PhyloCon recovered the motif at a rate comparable to that of our algorithm, and only marginally affected by the increasing noise in the data. PhyloCon performed well in retrieving the Fur motif, but with a recovery rate slightly sensitive to noise. Calculating the false positive discovery rate for PhyloCon illustrates that this algorithm is not sensitive to low signal to noise ratios (false positives ranging between 5.1 and 5.7) (Table 1). However, the absolute number of false positives is higher than that in the case of our methodology.

Regarding the quality of the motif models, the Fur motif model retrieved by PhyloCon seemingly outperforms our retrieved model both in sensitivity and specificity with hardly any effect for the noise in the data set. For the LexA motif, an optimal specificity was obtained but with mediocre sensitivity, indicating that the motif model, although frequently recovered, is often based on just two or three LexA instances.

It is important to emphasize that all the values obtained for the performance assessment of PhyloCon are biased to its favor in this case. One needs to note that in all the occasions when PhyloCon did not converge to a known motif, we retrieved the motif model from a cycle premature of convergence (for details see Methods: Benchmarking with other methodologies). Thus, slightly better sensitivities and recovery rates of PhyloCon over our methodology need to be interpreted in the light of this fact.

Again, our methodology proved to be more robust to noise than similarly based algorithms as seen in its ability to recover all the true motif models in the data set with very few false positives.

### Evaluation of the analysis flow on expression data

**Figure 4:**
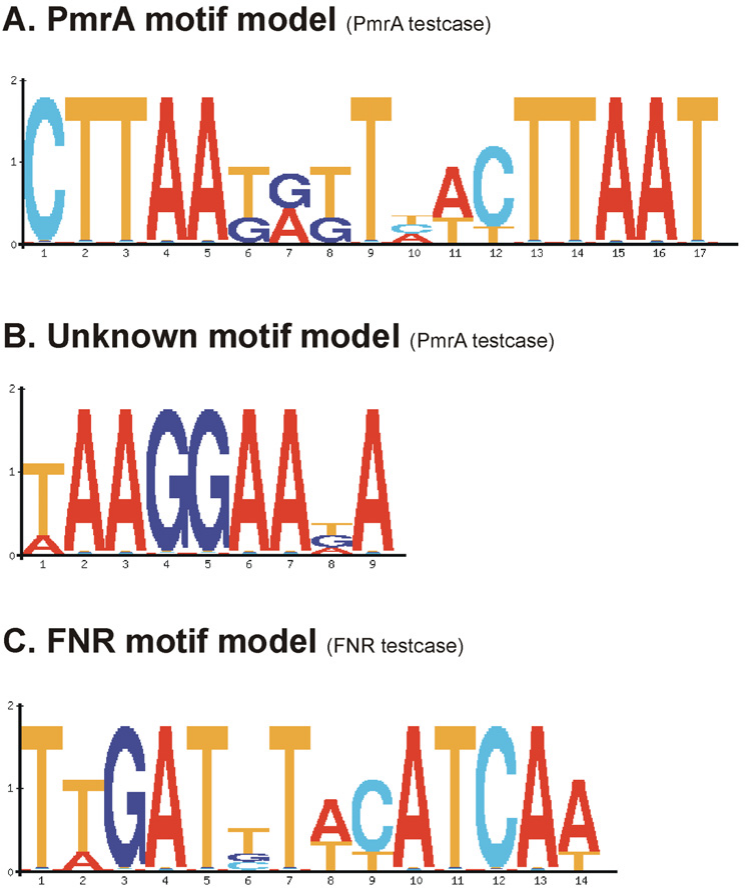
**Motif logos of the motif models resulting from the analysis of expression data**. A. PmrA motif model (PmrA testcase). B. Motif model of unknown regulatory motif (PmrA testcase). C. FNR motif model (FNR testcase).

Besides the test data sets, we applied our methodology on two data sets derived from genome wide expression studies. A first data set consisted of a list of 47 differentially expressed genes between a constitutive and a null *pmrA *mutant of *S. typhimurium *[34]. The PmrAB two-component regulatory system is part of a multi-component feedback loop that acts as a major virulence regulator in *S. typhimurium*. The system itself is responsive to Fe^3+ ^and mild acid and senses Mg^2+ ^indirectly by communicating with the Mg^2+ ^sensitive PhoPQ system via PmrD. Applying our analysis flow on the intergenic sequences of these coexpressed genes (BlockSampler, followed by BlockAligner and clustering) resulted in the detection of two potential motifs. The first motif which was derived from the motif instances of the known PmrA regulated genes *udg*, *yfbE*, *yibD *and *yjdB*, corresponded to the consensus of the biologically validated PmrA motif (consensus CTTAA-N_5_-CTTAA) [23,35,36]. The motif logo is shown in figure 4.A (details about motif instances: Additional file 2 or [33]) Screening the intergenic regions of the remaining differentially expressed genes from the input set with this motif model, resulted in the detection of six additional potential PmrA targets. Two of these six genes (*S. typhimurium *(*STM1269 *and *ais*) did not contain an ortholog in *E. coli *or *Y. pestis*, and in the four remaining genes (*ybjG, ygiW, yijP *and *yegH*) the PmrA motif was not conserved in the orthologs of *E. coli *or *Y. pestis*, explaining the reason these motif instances could not be recovered initially unless by screening.

The (direct or indirect) regulation of eight of these reported ten genes by PmrAB is supported by evidence from previous studies. Seven of these genes – *ais (pmrG), yjdB (pmrC), yfbE (pmrH), ugd *(*udg, pmrE, pagA), yibD*, *ybjG *(*mig-13*) and *STM1269 *(*aroQ*) – are known members of the PmrAB regulon that were confirmed in different experiments [23,34-38]. The last gene, *yijP (STM4118)*, was also discovered in an *in silico *screening of this same microarray data by Tamayo *et al*. [34].

We also report two new potential PmrA targets, *ygiW *and *yegH*, that were not recovered by Tamayo *et al *[34]. This could be due to the more stringent threshold they used when defining differentially expressed genes, than ours (see above).

In addition to the well known PmrA motif, we predicted yet an uncharacterized motif (consensus (T/A)AAGGAAnA) (figure 4B) which was based on conserved instances present in *tufA*, *yihT *and *STM2186 *(Additional file 2 or [33]). Screening the remaining differentially expressed genes with the motif model corresponding to this motif resulted in the discovery of motif instances in two additional genes (*sopD *and *STM1472*). No similarity between this motif and any of the motifs in the existing databases could be found (RegulonDB [39]).

The second data set was derived from the study of Salmon *et al*. [40]. *E. coli *and related species respond to oxygen depletion by switching to anaerobic respiration. This transition is mainly controlled by the global regulator FNR [41]. Salmon *et al*. [40] performed genome wide expression profiling experiments to identify genes differentially expressed in response to oxygen and controlled by FNR. Applying our methodology to 83 differentially expressed genes derived from their study resulted in the detection of a motif model corresponding to the well known FNR motif (motif logo: figure 4.C, details of motif instances: Additional file 2 and [33]). The model was based on conserved instances found in 4 known FNR regulated genes *narX*, *nirB, ndh *and *cydA *(RegulonDB). The instances present in three differentially expressed genes, marked as FNR regulated in RegulonDB (*cyoA, dcuC *and *frdA*) did not contribute to our FNR motif model. It seems like the degree of conservation of the motif among the respective orthologs of these genes was too low to be detected by our analysis flow. However, these genes could be retrieved in the screening step.

## Discussion

If we want to be able to get a complete view on the transcriptional network of an organism, revealing all regulatory motifs remains one of the main challenges [15]. Despite the fact that several motif detection algorithms have been developed and optimized, detection of regulatory motifs with low overrepresentation is still difficult using the common motif detection tools. Therefore we developed a tool that is able to retrieve regulatory motifs planted in a noisy environment i.e. only a very small subset of submitted intergenic regions contains the motif. In a first step of our method, large conserved regulatory regions (i.e. blocks) are identified in a set of orthologous intergenic regions (BlockSampler). After aligning all these conserved blocks with each other, blocks containing a shared regulatory motif are clustered. In a final step, screening of all input intergenic regions permits for the detection of those target genes where the motif was not conserved in its orthologs (if existing).

To test its reliability, we applied our methodology on a "golden standard" consisting of a set of randomly selected, non-related genes among which known targets of the Fur, MetJ, LexA and PhoP regulons were hidden. Although these known targets contained previously described motif instances for each of these regulators, their statistical overrepresentation in this test set was low (18% going down to 7% for Fur and LexA, 11% down to 4% for MetJ and PhoP (dyad motif)). When applying our methodology we found that for 3 out of the 4 hidden motifs the recovery rate was above 90% even in the presence of a high amount of noisy genes. For the Fur motif it was slightly lower (50% on average) because of the lower degree of conservation of the motif model (consensus score of 1.02 for the Fur motif versus 1.21, 1.27 and 1.26 for the MetJ, PhoP and LexA motif respectively). Notwithstanding this high recovery rate, the number of false positive motifs (i.e. motifs not corresponding to any of the motifs hidden in the data set) remained low (an average of 0.50 false positives per run when 10 random genes are added versus 0.80 when 50 random genes are added). The achieved quality of the retrieved motif models, reflected by the motif model sensitivity (the number of true instances contributing to the motif model) and specificity (the number of false positive instances degrading the motif model) depends on the characteristics of the motifs; well conserved, non-dyad motifs such as the LexA and MetJ motifs are seemingly easy to retrieve and have a high quality; dyad motifs (PhoP and Fur), although having high specificity (above 80%), their sensitivity was lower than in the case of non-dyad motifs, especially for the PhoP dyad. In the case of the PhoP motif, the low sensitivity is due to the fact that it also occurs as a single half site. The score of the alignment of the single half site with the dyad falls below the thresholds we used and therefore these single sites could not be recovered by our methodology.

To assess the influence of taking into account orthology information in addition to information from coexpression, we applied our methodology to a combined data set of orthologous and coexpressed genes and compared its results to those obtained by applying AlignACE, which is a motif detection tool based on Gibbs sampling, to the coexpression data only. Our methodology clearly outperformed AlignACE in recovering the true motifs in the data set. In addition, the performance of AlignACE was seriously influenced by increasing noise in the data, in contrast to what is observed with our methodology. These results indicate that by the incorporation of orthology information, the retrieval of motifs with weak overrepresentation (ranging between 11% and 4%) becomes possible.

In addition to the above mentioned advantage of using the combined sources of information of coexpression and orthology, an extra value can be deduced. When using phylogenetic footprinting alone on orthologous gene sets of closely related species sharing a large part of the regulatory mechanism, long conserved blocks are detected rather than small distinct motifs [23]. Because this complicates the delineation of specific motifs, the sequential use of BlockSampler (utilizing orthology information) and BlockAligner (utilizing coexpression information) allows for an improved delineation of individual regulatory motifs from those long conserved regions across orthologs.

We also compared our methodology to PhyloCon, as it has a similar strategy as ours. PhyloCon identifies conserved regulatory regions (profiles, called blocks in our study) in sets of orthologs based on the Wconsensus program. Subsequently, these profiles are merged between different sets of orthologs using a greedy algorithm, in order to detect a common regulatory motif.

PhyloCon was originally developed to detect motifs in a set of coexpressed genes, containing only one motif. The test sets used in the original paper usually contain a single motif per data set. As was noted by the authors, PhyloCon would perform better on less conserved motifs. Indeed, our results show that PhyloCon had a better performance in retrieving the more degenerated Fur motif than the well conserved LexA, MetJ and PhoP motifs. Despite this, the overall recovery rate was lower than that of our methodology in noisy data sets containing more than one motif. The MetJ and PhoP motifs were not recovered at all. Although the authors stated that their methodology can retrieve regulatory regions present in only a small set of genes (e.g. MetJ), in our experience PhyloCon fails to retrieve such motifs in a noisy context. In addition, as PhyloCon proceeds cycle per cycle, where in each cycle a new motif instance is added to the motif model, the authors suggest that the motif model building process be stopped before convergence in order to detect more motifs in a single dataset. However, they do not provide a clear stop criteria or heuristics to retrieve the optimal model. In order not to bias our results towards a sub-optimal stop criterion, we checked at each cycle whether a motif model was found that matched our test data set. If found during at least one cycle, the motif was considered "recovered". Note that this approach is feasible when one knows which motif to look for, but not in the case when a novel motif is searched for. This approach led to the bias of the assessment results to the favor of PhyloCon, as seen in the higher recovery rate, sensitivity and specificity acquired for PhyloCon over our method.

As a final proof of concept, we also applied our methodology to two gene sets derived from genome wide expression profiling experiments. Firstly, out of 47 differentially expressed genes between constitutive and null *pmrA *mutants of *S. typhimurium *[34], we could retrieve the expected PmrA motif model [23,35,36], and predict the presence of two new putative PmrA targets, *ygiW *and *yegH*. We also predicted a yet uncharacterized motif of the consensus (T/A)AAGGAAnA that was based on conserved instances in the genes *tufA, yihT *and *STM2186*. In a second test, using a list of 83 FNR regulated genes [40], we could retrieve the FNR motif model based on 4 genes containing a clear FNR regulatory motif.

## Conclusion

Conclusively, we have developed an approach that can reliably identify multiple regulatory motifs lacking a high degree of overrepresentation in a set of coexpressed genes (motifs belonging to sparsely connected hubs in the regulatory network) by exploiting the advantages of using both coexpression and phylogenetic information. Through comparing our methodology to two other motif detection programs, we show the robustness of our implementation. As a proof of concept, analysis of genome wide expression data with our methodology successfully retrieves the present regulatory motifs.

## Methods

### Input data sets

The selection of intergenic regions and the construction of species specific background models, relied on modules implemented in INCLUSive [30]. Intergenic regions are defined as the non-coding parts between two coding sequences. The regions used in this study were derived from the following genomes: *Escherichia coli *K12 [GenBank: NC_000913], *Escherichia coli *plasmid R721 [GenBank: NC_002525], *Escherichia coli *O157:H7 EDL933 [GenBank: NC_002655], *Escherichia coli *O157:H7 [GenBank: NC_002695], *Escherichia coli *CFT073 [GenBank: NC_004431], *Escherichia coli *O157:H7 plasmid pO157 [GenBank: NC_002128], *Escherichia coli *plasmid pB171 [GenBank: NC_002142], *Shigella flexneri *2a str. 301 [GenBank: NC_004337], *Shigella flexneri *virulence plasmid pWR501 [GenBank: NC_002698], *Salmonella typhimurium *LT2 [GenBank: NC_003197], *Salmonella typhimurium *LT2 plasmid pSLT [GenBank: NC_003277], *Salmonella Typhi *CT18 [GenBank: NC_003198], *Salmonella Typhi *CT18 plasmid pHCM1 [GenBank: NC_003384], *Salmonella Typhi *CT18 plasmid pHCM2 [GenBank: NC_003385], *Yersinia pestis *CO92 plasmid pPCP1 [GenBank: NC_003132], *Yersinia pestis *CO92 [GenBank: NC_003143], *Yersinia pestis *CO92 plasmid pCD1 [GenBank: NC_003131], *Yersinia pestis *KIM [GenBank: NC_004088], *Yersinia pestis *CO92 plasmid pMT1 [GenBank: NC_003134].

Close homologs (either orthologs or paralogs) were identified as described in Marchal *et al*. [23].

For benchmarking, different test data were compiled consisting of a core set of genes with known motifs (PhoP, LexA, Fur and MetJ) supplemented with sets of random genes varying in number and composition. A core set of genes with known binding sites was selected based on the RegulonDB database [39] for respectively LexA, Fur, and MetJ. Genes containing known PhoP motifs were selected from Monsieurs *et al*. [42]. The composition of the core gene set is heterogeneous in terms of the number of instances and conservation of each motif. An overview of this composition is given in figure 2. For MetJ (3 genes) and LexA (5 genes), the motif instances where conserved in all the species used in this study. For 3 out of the 5 Fur regulated genes and 1 out of the PhoP regulated genes, a motif instance was not present in *Y. pestis*. Starting from this core set, different test data were generated by gradually adding an increasing number of random genes. To this end, genes having an intergenic sequence larger than 100 nucleotides and a sufficient number of orthologs in the organisms of interest were selected randomly from the *Salmonella *genome. By sampling ten times respectively 10, 20, 30, 40 and 50 random genes and adding these to the core gene set, a total of 50 test sets was created.

For the construction of the PmrA data set, the data corresponding to an experiment described by Tamayo *et al*. [34] were downloaded from the Stanford Microarray Database [43]. In their analysis, Tamayo *et al *compared the mRNA expression between PmrA-constitutive and PmrA-null strains at two different time points (early- and mid-logarithmic phase of growth). As input data set, we selected two times 40 genes out of this microarray results that were most up- or down-regulated respectively in both conditions. Notice that we used a less stringent threshold than Tamayo *et al*. [34] who only selected 41 genes that exhibited a minimal fold change of 2 at both time points. After elimination of those genes with in an intergenic region smaller than 50 nucleotides, only 47 out of these 80 genes were retained. For the application of our methodology on data sets derived from genome wide expression studies, we relied on the data previously published in Salmon *et al*. [40] to build the FNR data set. All 125 genes assigned by Salmon *et al*. to a cluster affected by a mutation of *fnr *(i.e. 6 out of the 8 different clusters), were combined and used as input data for our methodology. From these 125 genes, 83 genes with an intergenic region longer than 50 nucleotides were retained.

### Analysis flow

#### Step 1: Detecting conserved blocks with BlockSampler

BlockSampler is based on the original Gibbs sampling algorithm of MotifSampler. Briefly, the Gibbs sampling procedure starts by searching for a motif shared by at least 2 sequences and having one occurrence in the reference sequence (the sequence of interest, see below). After convergence, short motif seeds are identified. The identification of these seeds is predicated on the log likelihood score [2]. This score depends on the degree of conservation of the motif and the number of instances detected in the species. Thus, the more species in which the motif is conserved and the higher its degree of conservation, the higher is its corresponding score. High scoring seeds are subsequently extended using a simple protocol: if the consensus score over a 5-nt region adjacent (upstream or downstream) to the current motif seed exceeds a given threshold the motif is extended with one nucleotide (in that direction). Detected conserved intergenic regions (i.e. blocks) of variable length are eventually reported. To select the most promising hits from the output of BlockSampler, we designed a score that is independent of block sequence length, but increases with the degree of conservation of the motifs. This normalized consensus score is appropriate because short motifs have a higher chance of resulting in a high consensus score. Normalization was done by recalculating the consensus scoring according to the following formula: Cs_ad _= (L/L+E) Cs, where Cs_ad _is the normalized consensus score, L is the length of the conserved block, E is an empirical factor (set to 6) and Cs the consensus score. Different empirical factors were tested on different data sets, and 6 appeared to give the best balance between block sequence length and conservation. Depending on the interest of a particular study, the empirical factor can be enlarged to favor larger blocks. Blocks are then ranked according to this normalized consensus score

BlockSampler requires six user-defined parameters: 1) the definition of a reference sequence: the reference sequence is the sequence in which the presence of the conserved block is required (in our case, it was set to be *Salmonella typhimurium*); 2) the number of runs: as BlockSampler is based on Gibbs sampling the algorithm should be repeatedly applied on the same input set (set at 100 runs); 3) strand: only the plus strand is searched; 4) prior: set at default value of 0.2; 5) threshold of the consensus score: only blocks exceeding a consensus score of 1.3 are retained; 6) minimal motif length: minimal width of the block is set to 8.

As is the case with other Gibbs sampling based motif detection procedures, the same block can be detected several times over the different runs of the algorithm. To compile a list of non redundant blocks, blocks overlapping for more than 75% were grouped. From each set of overlapping blocks, the one displaying the highest (normalized) consensus score was chosen as representative and retained for further analysis. Each block is represented by a motif model, in the form of a frequency matrix.

#### Step 2: Aligning conserved blocks using BlockAligner

The algorithm uses a local ungapped alignment strategy based on dynamic programming to mutually compare conserved blocks represented by their respective motif models (frequency matrices M1 and M2). The following additive scoring scheme is used: the total alignment score of two motif models is the sum of the individual column scores. A column score (S) is defined as the distance between two aligned columns of the frequency matrices. As a measure, the Kullback-Leiber distance between two probability distributions is used, since the columns of a motif model can be considered to be the parameters of multinomial distributions. To make the scoring scheme compatible with dynamic programming, matching columns should score positively and non-matching columns negatively. Therefore the minimal match value of the Kullback-Leiber distance T was introduced. T is a user defined parameter that determines the stringency of the alignment. Columns with a score below T receive a negative score, while columns with a score above T receive a positive score. As a result, the following score of the alignment of two columns, i and j of two conserved blocks (represented by the frequency matrices M1 and M2) can be calculated:

S(i,j)=T−∑b=ATM1b,i*log⁡M1b,iM2b,j+M2b,j*log⁡M2b,jM1b,i
 MathType@MTEF@5@5@+=feaafiart1ev1aaatCvAUfKttLearuWrP9MDH5MBPbIqV92AaeXatLxBI9gBaebbnrfifHhDYfgasaacH8akY=wiFfYdH8Gipec8Eeeu0xXdbba9frFj0=OqFfea0dXdd9vqai=hGuQ8kuc9pgc9s8qqaq=dirpe0xb9q8qiLsFr0=vr0=vr0dc8meaabaqaciaacaGaaeqabaqabeGadaaakeaacqWGtbWucqGGOaakcqWGPbqAcqGGSaalcqWGQbGAcqGGPaqkcqGH9aqpcqWGubavcqGHsisldaaeWbqaaiabd2eanjabigdaXmaaBaaaleaacqWGIbGycqGGSaalcqWGPbqAaeqaaaqaaiabdkgaIjabg2da9iabdgeabbqaaiabdsfaubqdcqGHris5aOGaeiOkaOIagiiBaWMaei4Ba8Maei4zaC2aaSaaaeaacqWGnbqtcqaIXaqmdaWgaaWcbaGaemOyaiMaeiilaWIaemyAaKgabeaaaOqaaiabd2eanjabikdaYmaaBaaaleaacqWGIbGycqGGSaalcqWGQbGAaeqaaaaakiabgUcaRiabd2eanjabikdaYmaaBaaaleaacqWGIbGycqGGSaalcqWGQbGAaeqaaOGaeiOkaOIagiiBaWMaei4Ba8Maei4zaC2aaSaaaeaacqWGnbqtcqaIYaGmdaWgaaWcbaGaemOyaiMaeiilaWIaemOAaOgabeaaaOqaaiabd2eanjabigdaXmaaBaaaleaacqWGIbGycqGGSaalcqWGPbqAaeqaaaaaaaa@6AED@

S(i,j) will be equal to T if column i and j of motif models M1 and M2 respectively, are exactly the same.

As a biological motif is often "gapped" i.e. consisting of conserved nucleotides intersected by some non-conserved nucleotides, we introduced a small non-match penalty. Remark that this is different from a "gap score" in alignment algorithms [29,44], as insertions and deletions are not explicitly modelled (we use a local ungapped alignment).

This leads to the following scheme for the alignment matrix:

A(i,j) = max(0,A(i-1,j-1) + S(i,j),A(i-1,j-1) – NonMatchScore) for i>1 && j>1

A(i,1) = max(S(i,1),0)

A(1,j) = max(S(i,1),0)

In our setup, BlockAligner was used with the following parameter set: T value = 0.40; minimal length of the reported common motif = 6 nucleotides.

To assess the significance of the results, the alignment procedure was repeated 100 times on the same motif models (M1 and M2) after randomly shuffling their columns. The distribution of the scores obtained by aligning these randomly shuffled motif models was used to estimate the parameters of an extreme value distribution. This background distribution allowed obtaining a p-value for the genuine alignment (i.e. assessing the probability of obtaining by coincidence the score observed when aligning the unshuffled blocks). Blocks with a p-value below 0.001 were considered significant.

#### Step 3: Clustering conserved blocks and delineating regulatory motifs

Conserved blocks shared by different orthologous gene sets were grouped using a graph based clustering algorithm TribeMCL [32]. Nodes of the graph represent conserved blocks and edges represent the quality of the alignment between these conserved blocks. We used the -log_10 _of the p-value of the pairwise alignments (see previous step) as weight measure for the edges. To prevent inflating spurious relations between blocks based on low scoring alignment scores, only alignments with a p-value lower than 0.001 were taken into account.

Based on the pairwise alignment scores between the conserved blocks grouped within the same cluster, a multiple alignment is created, which is subsequently converted into a frequency matrix. Such matrix representing the multiple alignment of conserved blocks in a cluster can be seen as a model of the average motif that is conserved in the intergenic region of several sets of orthologous genes and to which all orthologous genes from the original orthologous sets of the cluster contribute. This multiple alignment was converted in a frequency matrix. From this frequency matrix, the minimal regulatory motif was defined as the 1) the region conserved in at least three reference genes, 2) with a minimal length of 6 nucleotides and 3) with a consensus score higher than or equal to 1.10. This minimal motif is extended with additional motif positions in both directions until the consensus score drops below the threshold of 1.10.

To construct a motif model specific for the reference species, the multiple alignment is based only on the motif instances contributing to the blocks that originate from the reference species.

#### Step 4: Genome wide screening for additional targets

Screening of promoter regions with the obtained motif models is performed using MotifLocator [23,45]. The cut-off value for a screening was derived based on the lowest MotifLocator score of known target genes of the corresponding regulatory protein.

### Benchmarking with other methodologies

#### Running AlignACE

AlignACE can be obtained at the AlignACE website [46]. AlignACE is a Gibbs Sampling algorithm for detecting regulatory motifs that are overrepresented in the promoter regions of a set of potentially coregulated genes. Therefore, the test sets used for AlignACE only contained information from coexpressed genes i.e. the intergenic regions of the reference genes from *S. typhimurium *sharing a similar motif. Orthologous information was not explicitly used. We run AlignACE with default parameter setting except that we give the GC content of species from which the intergenic regions are used as input (0.52 for *S. typhimurium*). AlignACE returns series of motif models that are overrepresented in the input promoter regions.

#### Running PhyloCon

PhyloCon uses the same information sources as our methodology [27]. This algorithm also starts from a set of genes that are potentially co-regulated (e.g. derived from microarray data) and uses orthologous information to detect novel regulatory motifs. This two-step procedure starts with aligning orthologous intergenic sequences and creating position specific scoring matrices (called profiles) based on the Wconsensus program [5]. Then PhyloCon compares these profiles generated from different genes and identifies the common regions in these profiles using a greedy approach. Because PhyloCon is optimised to use the same sources of information (both coexpression and orthology) as our methodology, the same test data sets could be used. When running PhyloCon (downloaded from [47]), the number of standard deviations was set between 0.5 and 2, but this only marginally affected the results. The way PhyloCon works is that the motif model grows cycle per cycle. In each cycle a new motif instance is added to the motif model. In the original article no clear stop criteria or heuristics were provided, so it is difficult to decide at which cycle an optimal motif model is detected. By screening all different cycles of the PhyloCon runs, we looked for runs of which at least one cycle shows a match to one of the known motif models. In a single run, more than one cycle can contain a motif of interest. For each particular motif the cycle that shows the highest combined sensitivity specificity score for the corresponding motif model was used for the calculation of the sensitivity, specificity and false positives.

#### Test Run

Each test run consisted of applying one of the specified algorithms to 10 test sets of similar composition (i.e. same number of random genes is added). The recovery rate of detecting a particular motif was defined as the percentage of test sets in which this motif could be recovered. For instance, if the LexA motif model was found in 6 of the 10 test sets each of which contained LexA regulated genes, the performance was defined to be 60%.

The exact content of a test run depends on the specificities of the algorithm applied. One test run of the methodology presented in this study was defined as applying the complete procedure on the test set (100 runs of BlockSampler, BlockAligner, Clustering, motif delineation). A single test run of PhyloCon or AlignACE was defined as running once the algorithm on the test set (see running PhyloCon and Running AlignACE).

#### Performance evaluation

To evaluate the performance of the different motif detection algorithms, we reported 1) the average recovery rate and 2) the average number of false positives. The average recovery rate reports how many times a motif model corresponding to a motif, known to be present in the test sets, was found on average in the test runs on different test sets of the same composition. 2) the average number of false positives reports how many times a motif model not corresponding to any motif known to be present in the test sets, was found on average in the test runs on different data sets of the same composition. Motifs known to be present in the data sets were represented by a benchmark of curated motif models extracted from the RegulonDB database [39]. A detected motif model was considered identical to a benchmark motif model when the Kullback-Leiber distance (as implemented in MotifComparison) between the two motif models was lower than 0.65 (default parameter); otherwise it was considered as a false positive. The calculation of the number of false positives for AlignACE and PhyloCon was done as follows: all motif models that were returned from the AlignACE algorithm, were aligned with the benchmark motif models. If such a motif model did not show similarity to any of the four motifs known to be present in the test sets, this motif model was regarded a false positive. For PhyloCon, no clear stop criterion is described. For that reason, we only took into account those cycles for which a hit with a benchmark motif model was detected. All motifs in these cycles that did not show a match with a benchmark motif model were treated as a false positive.

#### Motif quality

To represent the quality of the obtained motif models, we calculated motif model sensitivity and specificity using the following definitions:

SENS=TPTP+FN×100%
 MathType@MTEF@5@5@+=feaafiart1ev1aaatCvAUfKttLearuWrP9MDH5MBPbIqV92AaeXatLxBI9gBaebbnrfifHhDYfgasaacH8akY=wiFfYdH8Gipec8Eeeu0xXdbba9frFj0=OqFfea0dXdd9vqai=hGuQ8kuc9pgc9s8qqaq=dirpe0xb9q8qiLsFr0=vr0=vr0dc8meaabaqaciaacaGaaeqabaqabeGadaaakeaacqWGtbWucqWGfbqrcqWGobGtcqWGtbWucqGH9aqpdaWcaaqaaiabdsfaujabdcfaqbqaaiabdsfaujabdcfaqjabgUcaRiabdAeagjabd6eaobaacqGHxdaTcqaIXaqmcqaIWaamcqaIWaamcqGGLaqjaaa@3FDD@

where TP is the number of true positives motif instances (i.e. motif instances known to be present in the data set that contributed to the detected motif model) and FN is the number of false negatives (i.e. motif instances known to be present in the data set that did not contribute to the detected motif model).

For definition of the specificity (SPEC), we used:

SPEC=TPTP+FP×100%
 MathType@MTEF@5@5@+=feaafiart1ev1aaatCvAUfKttLearuWrP9MDH5MBPbIqV92AaeXatLxBI9gBaebbnrfifHhDYfgasaacH8akY=wiFfYdH8Gipec8Eeeu0xXdbba9frFj0=OqFfea0dXdd9vqai=hGuQ8kuc9pgc9s8qqaq=dirpe0xb9q8qiLsFr0=vr0=vr0dc8meaabaqaciaacaGaaeqabaqabeGadaaakeaacqWGtbWucqWGqbaucqWGfbqrcqWGdbWqcqGH9aqpdaWcaaqaaiabdsfaujabdcfaqbqaaiabdsfaujabdcfaqjabgUcaRiabdAeagjabdcfaqbaacqGHxdaTcqaIXaqmcqaIWaamcqaIWaamcqGGLaqjaaa@3FC5@

where TP is defined as stated above and FP is the number of false positives (i.e. motif instances not corresponding to any of the known motifs present in the data set contributing to the detected motif model). Remark that the definition of FP is dependent on the accuracy and completeness of the existing annotation in the motif databases.

### Software availability

BlockSampler and BlockAligner can be downloaded from our supplementary website [33]. Stand-alone versions of BlockSampler and BlockAligner and their corresponding help files are also provided as additional files (BlockSampler: additional file 3, help file BlockSampler additional file 4, BlockAligner additional file 5, help file BlockAligner additional file 6).

## Authors' contributions

PM carried out the computational analysis and wrote the manuscript. GT developed BlockSampler and BlockAligner. AF helped to draft the manuscript. SD and JV helped with biological analysis of the results. BDM critically read the draft. KM conceived of the study, coordinated the work and helped to draft the manuscript. All authors read and approved the final manuscript.

## Description additional data files

The following additional data are available with the online version of this paper. Additional file 1 contains the table describing the performance of our methodology, AlignACE and PhyloCon. Additional file 2 contains all details about the instances of the detected regulatory motifs in the biological cases. Stand-alone versions of BlockSampler and BlockAligner and their corresponding help files are also provided as additional data files (BlockSampler: additional file 2, help file BlockSampler additional file 3, BlockAligner additional file 4, help file BlockAligner additional file 5).

## Supplementary Material

Additional File 1 Contains the table describing the performance of our methodology, AlignACE and PhyloCon. Click here for file

Additional File 2 Contains all details about the instances of the detected regulatory motifs in the biological cases. Stand-alone versions of BlockSampler and BlockAligner and their corresponding help files are also provided as additional data files (BlockSampler: Click here for file

Additional File 3 Click here for file

Additional File 4 Click here for file

Additional File 5 Click here for file

Additional File 6 Click here for file

## Figures and Tables

**Table 1:** Number of false positives. Number of false positives on average per run obtained by applying the different algorithms on the respective test sets. Number of false positive motif models is given for each number of added random genes (# Random) to the initial test set

**# Random **	**New methodology**	**AlignACE**	**PhyloCon**
10	0.50	17.1	5.4
20	0.60	18.1	5.4
30	0.80	17.4	5.1
40	0.50	18.3	5.7
50	0.80	16.9	5.3
