# Supplementary material for: More robust detection of motifs in coexpressed genes by using phylogenetic information
Source: BMC Bioinformatics. 2006 Mar 20;7:160. doi: 10.1186/1471-2105-7-160 (PMC1525208; doi:10.1186/1471-2105-7-160)
Supplement: Additional File 1 — Contains the table describing the performance of our methodology, AlignACE and PhyloCon. [file 1471-2105-7-160-S1.pdf]

Supplementary data file 1:

Performance of the different motif detection algorithms on the different test data sets: Recovery rate (rec) and the sensitivity (sens) and specificity (spec) for the different motif models. In the last column, the average value taken over the different motif models is displayed. A) performance of the new methodology B) performance of AlignACE C) performance of PhyloCon.

Table 1.A

	**Fur**			**MetJ**			**LexA**			**PhoP**			**Overall**		
# Randomgenes	*Rec*	*Sens*	*Spec*	*Rec*	*Sens*	*Spec*	*Rec*	*Sens*	*Spec*	*Rec*	*Sens*	*Spec*	*Rec*	*Sens*	*Spec*
10	40	90	100	100	100	100	90	89	100	90	58	82	80	84	95
20	50	80	90	100	100	100	100	82	100	80	58	91	83	80	95
30	50	84	97	90	100	97	90	91	96	100	56	92	83	83	95
40	50	80	79	90	100	100	90	76	96	100	56	89	83	78	91
50	60	56	83	90	100	88	90	88	95	80	58	87	80	75	88

Table 1.B

	**Fur**			**MetJ**			**LexA**			**PhoP**			**Overall**		
# Randomgenes	*Rec*	*Sens*	*Spec*	*Rec*	*Sens*	*Spec*	*Rec*	*Sens*	*Spec*	*Rec*	*Sens*	*Spec*	*Rec*	*Sens*	*Spec*
10	40	100	49	0	-	-	20	100	53	0	-	-	15	100	51
20	30	100	51	0	-	-	10	100	68	0	-	-	10	100	59
30	20	100	45	0	-	-	10	100	61	0	-	-	8	100	53
40	10	100	54	0	-	-	10	100	54	0	-	-	5	100	54
50	10	100	48	0	-	-	0	-	-	0	-	-	3	100	48

Table 1.C

	**Fur**			**MetJ**			**LexA**			**PhoP**			**Overall**		
# Randomgenes	*Rec*	*Sens*	*Spec*	*Rec*	*Sens*	*Spec*	*Rec*	*Sens*	*Spec*	*Rec*	*Sens*	*Spec*	*Rec*	*Sens*	*Spec*
10	100	100	98	0	-	-	100	52	100	0	-	-	50	76	99
20	80	98	98	0	-	-	100	52	100	0	-	-	45	75	99
30	80	100	98	0	-	-	90	47	100	0	-	-	43	73	99
40	70	100	98	0	-	-	80	55	100	0	-	-	38	78	99
50	70	97	95	0	-	-	80	58	100	0	-	-	38	77	97
Overall	80	99	97	0	-	-	90	53	100	0	-	-	43	76	99
