# Supplementary material for: More robust detection of motifs in coexpressed genes by using phylogenetic information
Source: BMC Bioinformatics. 2006 Mar 20;7:160. doi: 10.1186/1471-2105-7-160 (PMC1525208; doi:10.1186/1471-2105-7-160)
Supplement: Additional File 2 — Contains all details about the instances of the detected regulatory motifs in the biological cases. Stand-alone versions of BlockSampler and BlockAligner and their corresponding help files are also provided as additional data files (BlockSampler: [file 1471-2105-7-160-S2.pdf]

Additional data file 2

This file contains all details of the about the instances of the detected regulatory motifs in the biological cases (PmrA and FNR). For every gene is indicated between brackets whether it contributed to the resulting motif model or whether is was recovered by the screening afterwards with the motif model. For every instance, the gene and accession number is given, the start and end position of the instance relative to the translation start, and the motif instance itself.

Biological testcase 1: PmrA

PmrA motif model

Gene	*Genome*	*Start*	*End*	*Instance*
udg (motif model)				
ECs2829	NC_002695	-163	-145	TGCTTAATATTAACTTAAT
udg	NC_003197	-163	-145	TGCTTAATATTAACTTAAT
ugd	NC_000913	-163	-145	TGCTTAATATTAACTTAAT
ugd	NC_002655	-163	-145	TGCTTAATATTAACTTAAT
ugd	NC_003198	-163	-145	TGCTTAATATTAACTTAAT
ugd	NC_004431	-163	-145	TGCTTAATATTAACTTAAT
yfbE (motif model)				
ECs3141	NC_002695	-85	-67	TTCTTAAGGTTAAGTTAAT
STY2527	NC_003198	-100	-82	TTCTTAATGTTAATTTAAT
YPO2422	NC_003143	-139	-121	TTCCTAAGGTTCATTTAAG
Z3511	NC_002655	-85	-67	TTCTTAAGGTTAAGTTAAT
b2253	NC_000913	-85	-67	TTCTTAAGGTTAAGTTAAT
y1917	NC_004088	-139	-121	TTCCTAAGGTTCATTTAAG
yfbE	NC_003197	-100	-82	TTCTTAATGTTAATTTAAT
yfbE	NC_004337	-118	-100	TTCTTAAGGTTAAGTTAAT
yfbE	NC_004431	-85	-67	TTCTTAAGGTTAAGTTAAT
yibD (motif model)				
ECs4493	NC_002695	-68	-50	GGCTTAATAGTTTCTTAAT
STY4088	NC_003198	-70	-52	GGCTTAATAGTTTCTTAAT
yibD	NC_000913	-68	-50	GGCTTAATAGTTTCTTAAT
yibD	NC_002655	-68	-50	GGCTTAATAGTTTCTTAAT
yibD	NC_003197	-70	-52	GGCTTAATAGTTTCTTAAT
yibD	NC_004431	-68	-50	GGCTTAATAGTTTCTTAAT
yjdB (motif model)				
ECs5096	NC_002695	-36	-18	AACTTAAGGTTGGCTTAAT
STY4492	NC_003198	-66	-48	TTCTTAAGGTTCACTTAAT
yjdB	NC_000913	-36	-18	AACTTAAGGTTGGCTTAAT
yjdB	NC_002655	-36	-18	AACTTAAGGTTGGCTTAAT
yjdB	NC_003197	-66	-48	TTCTTAAGGTTCACTTAAT
yjdB	NC_004337	-21	-3	AACTTAAGGTTGGCTTAAT
yjdB	NC_004431	-21	-3	AACTTAAGGTTGGCTTAAT
y1960	NC_004088	-451	-433	TCTTAAGGATTAACCTAAT
YPO2377	NC_003143	-48	-30	TCGATATTCTTTAGGTAAT
STM1269 (screening)				
STM1269	NC_003197	-63	-45	TACTTAATGTTATCTTAAT
aroQ	NC_003198	-63	-45	TACTTAATGTTATCTTAAT
ais (screening)				
ais	NC_003197	-65	-47	TGCGGAATTTCATTTTAAT
ybjG (screening)				
ybjG	NC_003197	-190	-172	ACTTTAAGGTTAATTTAAG
ybjG	NC_003198	-190	-172	ACTTTAAGGTTAATTTAAG
ygiW (screening)				
STY3353	NC_003198	-58	-40	TTGTTAAGGATTACCTTAT
ygiW	NC_003197	-58	-40	TTGTTAAGGATTACCTTAT
yijP (screening)				
yijP	NC_003197	-66	-48	TGCTTCAGATTCTTTTAAG
yijP	NC_003198	-66	-48	TGCTTCAGATTCTTTTAAG
yegH (screening)				
yegH	NC_003197	-262	-244	AGATTAATATTAAATGAAT
yegH	NC_003198	-262	-244	AGATTAATATTAAATGAAT

Unknown motif model

Gene	Genome	Start	End	Instance
tufA (motif model)				
ECs4190	NC_002695	-15	-7	TAAGGAATA
tufA	NC_000913	-15	-7	TAAGGAATA
tufA	NC_002655	-15	-7	TAAGGAATA
tufA	NC_003143	-15	-7	TAAGGAATA
tufA	NC_003197	-15	-7	TAAGGAATA
tufA	NC_003198	-15	-7	TAAGGAATA
tufB	NC_004088	-946	-938	TAAGCAATA
yihT (motif model)				
ECs4804	NC_002695	-15	-7	AAAGGAATA
yihT	NC_000913	-15	-7	AAAGGAATA
yihT	NC_002655	-15	-7	AAAGGAATA
yihT	NC_003197	-14	-6	AAAGGAAAA
yihT	NC_003198	-14	-6	AAAGGAAAA
yihT	NC_004337	-15	-7	AAAGGAATA
STM2186 (motif model)				
ECs3038	NC_002695	-14	-6	TAAGGAAGA
STM2186	NC_003197	-14	-6	TAAGGAAGA
STY2416	NC_003198	-14	-6	TAAGGAAGA
Z3401	NC_002655	-14	-6	TAAGGAAGA
b2146	NC_000913	-14	-6	TAAGGAAGA
yeiT	NC_004337	-14	-6	TAAGGAAGA
yeiT	NC_004431	-14	-6	TAAGGAAGA
sopD (screening)				
sopD	NC_003197	-14	-6	GAAGGAAAA
sopD	NC_003198	-14	-6	GAAGGAAAA
STM1472 (screening)				
STM1472	NC_003197	-9	-1	AAAGGAACA
STY1650	NC_003198	-9	-1	AAAGGAACA

Biological testcase 1: FNR

FNR motif model

Gene	*Genome*	*Start*	*End*	*Instance*
narX (motif model)				
ECs1727	NC_002695	-242	-230	TTGATATTTATCA
narX	NC_000913	-242	-230	TTGATATTTATCA
narX	NC_002655	-242	-230	TTGATATTTATCA
narX	NC_003143	-173	-161	GTGATTTATTTTA
narX	NC_003197	-282	-270	TTGATATAAATCA
narX	NC_003198	-281	-269	TTGATATAAATCA
narX	NC_004088	-149	-137	GTGATTTATTTTA
narX	NC_004337	-242	-230	TTGATATTTATCA
nirB (motif model)				
ECs4216	NC_002695	-73	-61	TTGATTTACATCA
nirB	NC_000913	-73	-61	TTGATTTACATCA
nirB	NC_002655	-73	-61	TTGATTTACATCA
nirB	NC_003143	-72	-60	TTGATTTACATCA
nirB	NC_003197	-73	-61	TTGATTTACATCA
nirB	NC_003198	-73	-61	TTGATTTACATCA
nirB	NC_004088	-72	-60	TTGATTTACATCA
nirB	NC_004337	-109	-97	TTGATTTACATCA
ndh (motif model)				
ECs1487	NC_002695	-151	-139	TTGATTAACATCA
YPO1617	NC_003143	-149	-137	TTGATATATATCA
ndh	NC_000913	-151	-139	TTGATTAACATCA
ndh	NC_002655	-151	-139	TTGATTAACATCA
ndh	NC_003197	-151	-139	TTGATGCACATCA
ndh	NC_003198	-151	-139	TTGATGCACATCA
ndh	NC_004088	-149	-137	TTGATATATATCA
ndh	NC_004337	-124	-112	TTGATTAACATCA
ndh	NC_004431	-124	-112	TTGATTAACATCA
cydA (motif model)				
ECs0768	NC_002695	-349	-337	TTGATATAAATCA
cydA	NC_000913	-346	-334	TTGATATTTATCA
cydA	NC_002655	-349	-337	TTGATATAAATCA
cydA	NC_003143	-315	-303	TTGTTCTAAATCA
cydA	NC_003197	-350	-338	TTGATTTTAATCA
cydA	NC_003198	-350	-338	TTGATTTTAATCA
cydA	NC_004088	-315	-303	TTGTTCTAAATCA
cydA	NC_004337	-346	-334	TTGATATTTATCA
cydA	NC_004431	-349	-337	TTGATATAAATCA
cyoA (screening)				
ECs0486	NC_002695	-242	-230	TTGTTTTATTTCG
cyoA	NC_000913	-242	-230	TTGTTTTATTTCA
cyoA	NC_002655	-242	-230	TTGTTTTATTTCG
cyoA	NC_003197	-322	-310	TTTCTTTACACGA
cyoA	NC_003198	-322	-310	TTTCTTTACACGA
dcuC (screening)				
ECs0660	NC_002695	-104	-92	TTGATTTTAATCA
dcuC	NC_000913	-104	-92	TTGATTTTAATCA
dcuC	NC_002655	-104	-92	TTGATTTTAATCA
dcuC	NC_003197	-102	-90	GTGATTTTAATCA
dcuC	NC_003198	-102	-90	GTGATTTTAATCA
dcuC	NC_004431	-104	-92	TTGATTTTAATCA

frdA (screening)				
frdA	NC_003197	-192	-180	TTAATCAAAATCA
frdA	NC_003198	-192	-180	TTAATCAAAATCA
frdA	NC_004088	-190	-178	ATTCTTAATATCA
