# Supplementary material for: More robust detection of motifs in coexpressed genes by using phylogenetic information
Source: BMC Bioinformatics. 2006 Mar 20;7:160. doi: 10.1186/1471-2105-7-160 (PMC1525208; doi:10.1186/1471-2105-7-160)
Supplement: Additional File 4 [file 1471-2105-7-160-S4.doc]

# BlockSampler Helpfile

| **Overview** |
| --- |

1. Required arguments
2. Optional arguments
3. Output definitions
4. Background model description
5. Example
6. References

The **BlockSampler** is used to find conserved blocks in the upstream region of sets of orthologous genes.
Some basic remarks on the program:

- The program should be started from the command line. A full description of the required and optional arguments can be found below.
- The final results are printed either on STDOUT or in a file in GFF format.
- The block models found can be saved in a separate file using the **-m** swicth.
- On the STDERR you can monitor the progress of the program.

| **Required Arguments** |
| --- |

| **Switch** | **Argument** | **Description** |
| --- | --- | --- |
| -f | file | Input sequences in fasta format. There should be at least 2 sequences in the file. |
| -b | file | File containing a list of sequence ids and the names referring to the related background model files. Format description of this file can be found below. |
| -i | value | Defines the root sequence of the data set. This value should be similar to one of the identifiers of the sequences in the fasta file. |

| **Optional Arguments** |
| --- |

| **Switch** | **Argument** | **Description** |
| --- | --- | --- |
| -s | 0|1 | Select strand. (default plus strand). 0 is only input sequences, 1 include reverse complement. |
| -p | value | Sets prior probability of finding one instance of the block. This value allows the user to define the required characteristics of the block to search for. If the prior is set close to 0 then more conserved blocks are retrieved, increasing the prior will introduce more degeneracy into the block model. If the prior is set too small, it is possible that no block is found. Default = 0.2. |
| -w | value | Sets length of the initial seed of the block (default 8). |
| -r | value | Set number of times the BlockSampler should be repeated (default = 100). When using this option it is best to define a matrix file with the '-m' switch to store the block models generated at each repetition. This file can later be used to analyze the block models and select the best scoring models. |
| -t | value | Sets threshold to extend block length (a value between 0 and 2, default = 1.0). |

| **Output Description** |
| --- |

| **Switch** | **Argument** | **Description** |
| --- | --- | --- |
| -o | file | Sets the output file to save the results. The found block instances are written to this file in GFF format. Default the results are written to STDOUT. |
| -m | file | Sets the file name of the matrix file to store the retrieved block models. If not provided the matrices are not saved. This matrix file can be used to screen DNA sequences for instances of the retrieved blocks.  If you have done multiple runs (switch '-r'), you should use this matrix file to further analyze the results. |

| **Background Model Description** |
| --- |

In BlockSampler each orthologous intergenic sequence in the input data set is scored with its appropriate species-specific background model (structure is given below). In order to provide information about which sequence should be scored with which background model, a file containing links to the different background models is required (see parameter -b).

ECs3478|NC_002695 /path/to/your/backgrounddir/backgroundNC_002695_3.bg

YPO1105|NC_003143 /path/to/your/backgrounddir/backgroundNC_003143_3.bg

recN|NC_000913 /path/to/your/backgrounddir/backgroundNC_000913_3.bg

recN|NC_002655 /path/to/your/backgrounddir/backgroundNC_002655_3.bg

recN|NC_003197 /path/to/your/backgrounddir/backgroundNC_003197_3.bg

recN|NC_003198 /path/to/your/backgrounddir/backgroundNC_003198_3.bg

recN|NC_004337 /path/to/your/backgrounddir/backgroundNC_004337_3.bg

recN|NC_004431 /path/to/your/backgrounddir/backgroundNC_004431_3.bg

A background model is stored as an ascii text file using a well defined format. Below you can find an example of the first-order *Salmonella typhimurium* background model file. The file should always start with the word #INCLUSive at the first position of the file. Next, there are several lines describing the organism, data set and order of the background model. Finally the data itself is represented.

--

#INCLUSive Background Model v1.0

#

#Order = 1

#Organism = NC_003197

#Sequences = NC_003197intergenics.txt

#Path =

#

#snf

0.2904 0.2094 0.2098 0.2903

#oligo frequency

0.2904

0.2094

0.2098

0.2903

#transition matrix

0.3408 0.1832 0.1796 0.2964

0.2944 0.2182 0.2437 0.2437

0.2620 0.2697 0.2197 0.2486

0.2533 0.1886 0.2105 0.3476

--

You can get some pre-compiled background models at our Background Model download page [1]. To create your own background model you can use the program CreateBackgroundModel which you can find on the INCLUSive website [2].

| **Example** |
| --- |

Here is a step-by-step example on how to use the BlockSampler. The current version is a Linux version. To make sure that all the file specifications are clear, an example data set is provided as additional data file on the supplementary website [3], together with the background model files.

**1. Software installation**

The first step is the installation of the program. Download our software from the supplementary website [3]. If you save it, make it executable (chmod 755 BlockSampler) and make sure that the program is included in your path. You can test if it works by just typing BlockSampler at the prompt without any option.
The output should look like this:

ssh|pmonsieu>BlockSampler

Seed = 768594569

Usage: BlockSampler

Required Arguments

-f <fastaFile> Sequences in FASTA format

-b <bgFile> File containing a list of sequence ids and

background model file names.

-i <value> Defines the root sequence of the data set. Should

be similar to the identifier of the sequence in

the fasta file.

Optional Arguments

-s <0|1> Select strand. (default plus strand) 0 is only

input sequences, 1 include reverse complement.

-p <value> Sets prior probability of 1 motif copy. (default

0.2)

-w <value> Sets length of the motif (default 8).

-r <runs> Set number of times the MotifSampler should be

repeated (default = 1).

-t <value> Sets threshold to extend motif length (default =

1.0).

Output formatting Arguments

-o <outFile> Output file to write results (default stdout).

-m <matrixFile> Output file to write retrieved motif models.

Version 3.1 -- the bug fix release

Questions and Remarks: gert.thijs@esat.kuleuven.be

**2. Input Sequences**

Input sequence should be in fasta format. An example is provided on the supplementary website [3].

**3. Background Model**

For this example we use the third-order background model from the different gamma-proteobacteria. The intergenic sequence of *recN* is derived from *Salmonella typhimurium* and is thus scored by a *Salmonella typhimurium*-specific background model, namely backgroundNC_003197_3.bg. In a similar way, each ortholog is scored with its species-specific background model. How to download or create background models is explained above.

**4. Run BlockSampler**

We use the default parameters of BlockSampler except for

- **-o example.out>**   The output is written in a gff file
- **-m example.matrix>**   Block models are written to a matrix file
- **-t 1.3**   Here we augment the threshold of the consensus score. This will allow the algorithm to find stronger conserved blocks.
- **-r 100**  we 100 runs of the BlockSampler.

**Command line:** BlockSampler -f example.fasta -b example.bg -i recN\|NC_003197 -o example.out -m example.matrix -t 1.3 -r 100 >error.log

*Note that in this example the output is written on STDOUT and the STDERR is redirected to 'error.log'.*

#INCLUSive GFF File

#id: block_recN|NC_003197_1 consensus: TACGyCAGCCTCTTTACTGTATATAAAACCAGTTTATACTGTAywCAATwACAGTmATGG sequences: 8 instances: 8 cs: 1.57 ic: 1.51 ll: 562.83

ECs3478|NC_002695 BlockSampler misc_feature 5 64 1.56612e+32 + . id "block_recN|NC_003197_1"; site "TACGCCAGCCTCTTTACTGTATATAAAACCAGTTTATACTGTACACAATAACAGTAATGG";

YPO1105|NC_003143 BlockSampler misc_feature 6 65 4.57455e+24 + . id "block_recN|NC_003197_1"; site "CACCTCAGCTACTTTACTGTATATAAAACCAGTCTATACTGTGTTCAAATACAGACATGT";

recN|NC_000913 BlockSampler misc_feature 5 64 2.51525e+32 + . id "block_recN|NC_003197_1"; site "TACGCCAGCCTCTTTACTGTATATAAAACCAGTTTATACTGTACACAATAACAGTAATGG";

recN|NC_002655 BlockSampler misc_feature 5 64 1.2066e+32 + . id "block_recN|NC_003197_1"; site "TACGCCAGCCTCTTTACTGTATATAAAACCAGTTTATACTGTACACAATAACAGTAATGG";

recN|NC_003197 BlockSampler misc_feature 5 64 2.60825e+29 + . id "block_recN|NC_003197_1"; site "TACGTTAGCCTCTTTACTGTATAAAAAACCAGTTTATACTGTATTTAATTACAGTCATGG";

recN|NC_003198 BlockSampler misc_feature 5 64 2.68962e+29 + . id "block_recN|NC_003197_1"; site "TACGTTAGCCTCTTTACTGTATAAAAAACCAGTTTATACTGTATTTAATTACAGTCATGG";

recN|NC_004337 BlockSampler misc_feature 5 64 2.06734e+32 + . id "block_recN|NC_003197_1"; site "TACGCCAGCCTCTTTACTGTATATAAAACCAGTTTATACTGTACACAATAACAGTAATGG";

recN|NC_004431 BlockSampler misc_feature 5 64 8.62813e+31 + . id "block_recN|NC_003197_1"; site "TACGCCAGCCTCTTTACTGTATATAAAACCAGTTTATACTGTACACAATAACAGTAATGG";

Take a look at the example of the gff file 'example.out' and matrix file 'example.matrix' at the supplementary information [3]. The resulting files should look more or less like this.

| **References** |
| --- |
