# Supplementary material for: More robust detection of motifs in coexpressed genes by using phylogenetic information
Source: BMC Bioinformatics. 2006 Mar 20;7:160. doi: 10.1186/1471-2105-7-160 (PMC1525208; doi:10.1186/1471-2105-7-160)
Supplement: Additional File 6 [file 1471-2105-7-160-S6.doc]

# BlockAligner Helpfile

| **Overview** |
| --- |

1. Required arguments
2. Optional arguments
3. Output definitions
4. INCLUSive motif model format
5. Example
6. References

The **BlockAligner** uses a local ungapped alignment strategy based on dynamic programming to mutually compare conserved promoter regions (i.e. blocks) represented by their respective motif models. Some basic remarks on the program:

- The program should be started from the command line. A full description of the required and optional arguments can be found below.
- The final results are printed either on STDOUT or in a file in GFF format.
- On the STDERR you can monitor the progress of the program.

| **Required Arguments** |
| --- |

| **Switch** | **Argument** | **Description** |
| --- | --- | --- |
| -m | file | File containing the query motif models (in INCLUSive format). Format description of this file can be found below. |
| -d | file | File containing database of models (in INCLUSive format) with which all query motifs will be compared. Format description of this file can be found below. |

| **Optional Arguments** |
| --- |

| **Switch** | **Argument** | **Description** |
| --- | --- | --- |
| -t | value | Maximal distance between two motifs to be considered as the same motif (default 0.4). |
| -g | value | Gap score (default 0.4). Because a biological motif is often "gapped" (i.e. consisting of conserved nucleotides intersected by some non-conserved nucleotides, a small non-match penalty can be introduced (i.e. "gap score"). Remark that this is different from a gap score as insertions and deletions are not explicitly modeled (local ungapped alignment). |
| -w | value | Sets the minimal length of reported common motif (default 4). |
| -s | value | To assess the significance of the results, the alignment procedure can be repeated a number of times on the same motif model but after randomly shuffling their columns Based on the alignment scores of these randomly shuffled motif models, the parameters for an extreme value distribution are estimated. This permits to assign a p-value to the real alignment. The number of times the alignment procedure is repeated with randomly shuffled motif models, can be set with the number of shuffles. The higher this number, the more accurate the parameter estimation of the extreme value distribution. |

| **Output Description** |
| --- |

| **Switch** | **Argument** | **Description** |
| --- | --- | --- |
| -o | file | Sets the output file to save the results. Default the results are written to STDOUT. |
| -M | file | Sets the file name of the matrix file to store the common matrices between both blocks. If not provided the matrices are not saved. |

| **INCLUSive motif model format** |
| --- |

A INCLUSive motif model is stored as an ASCII text file using a well defined format. Below you can find an example of conserved blocks found in the intergenic regions of *recN* in *Salmonella typhimurium* and its orthologs. The file should always start with the word #INCLUSive at the first position of the file. Next, there are lines representing the BlockID, the score, the width and the consensus of the motif model respectively. Finally the data itself is represented, where each row represents one position in the motif model, and each column represents one of the 4 bases (A, C, G or T, in that order).

#INCLUSive Motif Model

#

#ID = block_recN|NC_003197_1

#Score = 562.831

#W = 60

#Consensus = TACGyCAGCCTCTTTACTGTATATAAAACCAGTTTATACTGTAywCAATwACAGTmATGG

0.0125109 0.128344 0.00868059 0.850465

0.970187 0.00863404 0.00868059 0.0124982

0.0125109 0.96631 0.00868059 0.0124982

0.0125109 0.128344 0.846647 0.0124982

0.0125109 0.607182 0.00868059 0.371627

0.0125109 0.726891 0.00868059 0.251917

0.970187 0.00863404 0.00868059 0.0124982

0.0125109 0.00863404 0.966357 0.0124982

0.0125109 0.96631 0.00868059 0.0124982

0.0125109 0.846601 0.00868059 0.132208

0.13222 0.00863404 0.00868059 0.850465

0.0125109 0.96631 0.00868059 0.0124982

0.0125109 0.00863404 0.00868059 0.970174

0.0125109 0.00863404 0.00868059 0.970174

...

...

| **Example** |
| --- |

Here is a step-by-step example on how to use the BlockAligner. The current version is a Linux version. To make sure that all the file specifications are clear, an example data set is provided as additional data file at our supplementary website [1].

**1. Software installation**

The first step is the installation of the program. Download our software from our supplementary website [1]. If you save it, make it executable (chmod 755 BlockAligner) and make sure that the program is included in your path. You can test if it works by just typing BlockAligner at the prompt without any option.
The output should look like this:

ssh|pmonsieu>BlockAligner

Seed = 2081726080

Usage: BlockAligner

Required Arguments

-m <matrixFile> File containing the query motif models.

-d <matrixFile> File containing database of models with which

all query motifs will be compared.

Optional Arguments

-t <value> Maximal distance between two motifs to be

considered as the same motif (default 0.4)

-g <value> Gap score (default 0.4)

-w <value> Minimal length of reported common motif

(default 4)

-s <value> Number of shuffles of blocks to assess

significance (default = 0)

-o <outFile> Output file to write results to.

-M <filename> File to write common matrices.

-v Version of MotifComparison

Version 3.1 -- the bug fix release

Questions and Remarks: Gert.Thijs@esat.kuleuven.be

**2. Input Matrices**

Input files containing the query matrix / matrices and the database matrices need to have the INCLUSive format (see above). An example of a database file and a query file are given at our supplementary website [1].

**3. Run BlockAligner**

We use the default parameters of BlockAligner except for

- **-o blockaligner.out**   The output is written to a text file
- **-M blockaligner.matrix**   Common matrices between query and database matrices are written to a matrix file
- **-w 6**   Common part between two overlapping matrices needs to be at least 8 nucleotids.
- **-s 100**  We perform 100 shuffles in order to assess a significance to each alignment with BlockAligner

**Command line:** BlockAligner -d database.matrix -m query.matrix -o blockaligner.out -M blockaligner.matrix -s 100 -w 8 >error.log

*Note that in this example the STDERR is redirected to 'error.log'.*

block_recN|NC_003197_76 72 5 block_lexA|NC_003197_24 97 76 21 3.3 +1 CTTTACTGTATAwAAAACCAG CATrAyTGTATATACACCCAG 0.0142371 0

block_recN|NC_003197_76 72 8 block_uvrB|NC_003197_13 88 64 19 3.7 -1 TACTGTATAwAAAACCAGT TACTGGATrAAAAAACAGT 3.52575e-05 0

block_recN|NC_003197_76 72 54 block_uvrB|NC_003197_78 87 27 9 1.7 -1 TTTTTCATA TTTTTAACA 0.674001 0

block_recN|NC_003197_76 72 54 block_uvrB|NC_003197_82 68 28 9 1.7 -1 TTTTTCATA TTTTTAACA 0.728504 0

block_recN|NC_003197_76 72 62 block_uvrB|NC_003197_92 80 58 9 2.13264 -1 ACAGGAAAA ACAGGAATA 0.0330056 0

block_recN|NC_003197_76 72 10 block_uvrD|NC_003197_1 26 6 18 3.4 +1 CTGTATAwAAAACCAGTT CTGTATAwATwCCCAGyT 8.71482e-05 0

block_recN|NC_003197_76 72 4 block_uvrD|NC_003197_32 8 0 8 1.4 +1 TCTTTACT TCTTCTCT 0.334046 0

block_recN|NC_003197_76 72 48 block_dinI|NC_003197_82 13 4 9 1.2 +1 TmATGGTTT TmsTrGmTT 0.29316 0

block_recN|NC_003197_76 72 6 block_dinI|NC_003197_89 38 1 27 5.1 +1 TTTACTGTATAwAAAACCAGTTTATAC TTAmCTGTATAwATAwCCAGTATATTC 1.09177e-06 0

This output contains the following information:

1. column 1: ID of the query matrix
2. column 2: lenght of the query matrix
3. column 3: start position of the overlapping part with the database matrix
4. column 4: ID of the database matrix
5. column 5: length of the database matrix
6. column 6: start position of the overlapping part with the query matrix
7. column 7: length of the overlapping part
8. column 8: score of the alignment
9. column 9: indicates whether overlap is found in direct version of database matrix or the reverse complement
10. column 10: consensus-site in the query matrix
11. column 11: consensus-site in the database matrix
12. column 12: p-value of the alignment (= 0 if number of shuffles s is 0)

Take a look at the example of the output file 'blockaligner.out' and overlapping matrix file 'blockaligner.matrix' on our supplementary website [1]. The resulting files should look more or less like this.

| **References** |
| --- |
